# "It's at a Time in Your Life When You Are Most Vulnerable": A Qualitative Exploration of the Financial Impact of a Cancer Diagnosis and Implications for Financial Protection in Health

**DOI:** 10.1371/journal.pone.0077549

**Published:** 2013-11-11

**Authors:** Aileen Timmons, Rachael Gooberman-Hill, Linda Sharp

**Affiliations:** 1 National Cancer Registry, Cork, Ireland; 2 Orthopaedic Surgery Research Group, School of Clinical Sciences, University of Bristol, Bristol, United Kingdom; Sapporo Medical University, Japan

## Abstract

Although cancer patients may incur a wide range of cancer-related out-of-pocket costs and experience reduced income, the consequences of this financial burden are poorly understood. We investigated: financial adjustments needed to cope with the cancer-related financial burden; financial distress (defined as a reaction to the state of personal finances); and factors that increase risk of financial difficulties. Two sets of semi-structured face-to-face interviews were conducted with 20 patients with breast, lung and prostate cancer and 21 hospital-based oncology social workers (OSWs) in Ireland, which has a mixed public-private healthcare system. Participants were asked about: strategies to cope with the cancer-related financial burden; the impact of the financial burden on the family budget, other aspects of daily life, and wellbeing. OSWs were also asked about patient groups they thought were more likely to experience financial difficulties. The two interview sets were analysed separately using a thematic approach. Financial adjustments included: using savings; borrowing money; relying on family and friends for direct and indirect financial help; and cutting back on household spending. Financial distress was common. Financial difficulties were more likely for patients who were older or younger, working at diagnosis, lacked social support, had dependent children, had low income or had few savings. These issues often interacted with one another. As has been seen in predominantly publically and predominantly privately-funded healthcare settings, a complex mixed public-private healthcare system does not always provide adequate financial protection post-cancer. Our findings highlight the need for a broader set of metrics to measure the financial impact of cancer (and to assess financial protection in health more generally); these should include: out-of-pocket direct medical and non-medical costs; changes in income; financial adjustments (including financial coping strategies and household consumption patterns); and financial distress. In the interim, cancer patients require financial information and advice intermittently post diagnosis.

## Introduction

There is a growing awareness of the financial impact that cancer can have both on newly diagnosed patients and those living with the condition [1,2]. Patients incur a wide range of direct medical and non-medical out-of-pocket costs, all of which have been documented in diverse healthcare settings [1–9] and, while the amounts incurred vary, and not all patients experience all costs, the total out-of-pocket costs that any one patient experiences may be substantial, either in absolute terms or in relation to their ability to meet them. Ability to meet extra costs is likely to depend on several factors notably income, and changes in this post-diagnosis have also been documented in diverse settings [10–14]. Individual or family circumstances at diagnosis (including financial commitments such as rent, mortgage, or loan payments, savings and other assets) are also important, as is the financial protection available (i.e. how far people are protected from the financial consequences of illness) from the legal, and health and social welfare systems. It has been noted that most health systems fail to offer citizens adequate financial protection because of insufficient financial risk pooling and prepayment mechanisms [15]; in general, in countries where out-of-pocket payments comprise a greater share of health funding, financial protection is worse.

Given the inadequacies of health-related financial protection it seems likely that some cancer patients may have to make financial adjustments to cope with their altered financial situation post-diagnosis. Furthermore, these financial adjustments may result in financial distress, which is defined as a reaction to the state of personal finances [16] resulting from one or more stressors or negative financial events [17]. Since financial distress is a subjective experience, individuals in the same financial situation may perceive different levels of this type of distress. Cancer-related financial adjustments and financial distress have received little attention, in part due to the fact that studies have focussed on particular aspects of the financial or economic impact such as specific out-of-pocket costs or impact on employment and income [18,19]. The lack of agreement on what is meant by, or should be included in, assessments of financial impact and standardised quantitative instruments to assess this has further delayed progress in this area [18]. 

Similar limitations have been highlighted in the broader area of financial protection in health.

Conventional frameworks for analysis focus on two financial protection indicators relating to the extent to which health payments are “catastrophic” (above a threshold percentage of household equivalent income) or “impoverishing” (pushing a household below the poverty line). Critics of these frameworks suggest that the measures of the consequences of inadequate financial protection are too narrow and are hence likely to underestimate the adverse consequences of inadequate financial protection [20]. Moreover, because these metrics are based on self-reported out-of-pocket medical expenditure, they effectively ignore the fact that poorer individuals often cannot afford to use health services and report very low or no health spending and are therefore (incorrectly) considered to be “protected” [15].

To date, the limited research on the financial impact of cancer has been conducted in countries with either a great reliance on private healthcare and a libertarian ideology (e.g. the USA which also has a small public system for those who can’t pay for private healthcare), or more publically-funded healthcare and an egalitarian ideology (e.g. Canada or the UK, where a small private system also functions) [2,4]. In most countries a mixture of libertarian and egalitarian systems operate [21], but little is known about the financial impact of cancer in such settings. The Irish system provides an interesting example. It has a complex structure and financing: both libertarian and egalitarian principles operate for different services and different individuals [21]. 

Qualitative methods have previously proved valuable for exploring and improving understanding of cancer patients’ experiences [4,6,22,23]. We therefore undertook qualitative interviews with cancer patients and key informants to investigate financial adjustments and financial distress in Ireland. This article reports some of the findings of a broader investigation of the financial impact of a cancer diagnosis for patients and their families. The specific aims of this part of the study were to explore: 1. the financial adjustments needed by patients to cope with their financial situation after a cancer diagnosis and 2. the impact of these on financial distress or well-being.

## Methods

### Setting

In Ireland, free publicly-funded healthcare is available to citizens who possess a “medical card”. At the time of the study, medical card eligibility was means-tested (To qualify for a medical card or GP visit card an individual’s weekly income must be below a certain figure for their family size. Cash income, savings, investments and property (except for their own home) are taken into account in the means test) for those aged <70 and universal for those aged ≥70; 30% of the population had a card [24]. Individuals without a medical card make co-payments for primary care and outpatient visits, and public hospital inpatient stays and pay full costs for prescription medications up to a maximum cost of €85/month with drug payment scheme. Around half the population have private medical insurance [25]; plans typically cover inpatient stays, but may not cover outpatient or primary care costs. Approximately 25% of the population have neither a medical card nor private health insurance, and approximately 5% have both. Employers are not legally obliged to provide sick pay, nor is there any protection against dismissal from work due to lengthy sickness absence (e.g. due to cancer). Ireland has a complex entitlement system: those who do not receive sick pay are eligible for statutory illness benefit if they have paid enough social insurance contributions, otherwise they can apply for other means-tested payments.

### Subjects and recruitment

Two groups of subjects were interviewed: people diagnosed with primary breast, prostate or lung cancer and hospital-based oncology social workers (OSWs). OSWs frequently provide patients with advice and assistance regarding financial matters (although this is not formally part of their remit), and so were considered key informants who could contribute expert knowledge of, and valuable insight into, the financial impact of cancer on patients and families. All 24 OSWs who worked with patients with breast, prostate or lung cancer were invited to participate. Patients were eligible if they were: aged ≥18; post-initial treatment; and had reported that they were experiencing “extra costs” or “financial difficulties” because of their cancer to a health professional (i.e. financial difficulties or extra costs were self-defined by patients). Patients receiving palliative care were excluded since their experiences of costs may be substantially different to those of other patients. Breast and prostate cancer are the most commonly diagnosed cancers in females and males respectively and lung cancer is the third most common cancer in both sexes. These cancers provided diversity in the sample and ensured that we could explore the experiences of patients: of both sexes; of varied ages and hence varied family circumstances and employment status; and who had a mixture of prognoses and treatment patterns. Moreover, since lung cancer is inversely related to social class and breast and prostate cancer risk is higher in the higher social classes [26], these sites provided diversity with respect to socio-economic circumstances. The interviewer (AT) collaborated with participating OSWs and oncology nurses to identify a purposive patient sample. OSWs and nurses were asked to identify potential participants on the basis of a range of ages and geographical spread (urban and rural dwellers from different locations across the country). They told eligible patients about the study and provided the study information sheet and a reply sheet. Then, if the patient indicated they were potentially willing to participate (returned reply slip), the OSW/nurse forwarded contact patient details to the interviewer. The interviewer telephoned patients, answered any questions, and arranged a suitable time and location for interview. Where possible this was scheduled to coincide with an outpatient visit. On the day of the interview, participants were given another opportunity to ask questions and once these had been answered satisfactorily, participants were asked to provide written consent just prior to starting the interview. Throughout recruitment, the interviewer liaised closely with the health professionals to maximise variation in the sample. Ethical approval was provided by the research ethics committee for each of the participating hospitals. The specific names of the hospitals and their research ethics committees are not provided to protect anonymity of the OSWs interviewed.

### Interviews

Interviews were conducted in 2007 (OSW interviews: February-March; patient interviews:

August-September).With the exception of two OSW interviews, all were face-to-face and audio-recorded (detailed notes were taken by AT during one face-to-face interview; the other was conducted by telephone and audio-recorded). OSW interviews took place at the individuals’ places of work. Patient interviews took place in a hospital or at a cancer support centre. Interview questions were structured using a topic guide. The OSW topic guide was developed from literature review and the results of analysis of welfare grants provided to patients by the Irish Cancer Society [27]. The patient topic guide was developed from literature review and analysis of the OSW interviews. Both patients and OSWs were asked about: strategies used to cope with any extra cancer-related costs or changes in income; impact of extra costs on the family budget and other aspects of daily life; and anything else they thought was important. Because OSWs have experience of talking to large numbers of patients about their financial situation, they were also asked about particular groups of patients that they thought were more likely to report the need for financial adjustments. Patients were asked about their socio-demographic characteristics and circumstances. Interviews lasted 30-120 minutes. Interviews were transcribed verbatim, checked for accuracy, and anonymised.

### Analysis

Recruitment to each group of subjects ceased once saturation was reached [28]. Saturation of data was reached after 17 OSW interviews and 15 patient interviews since no new themes or issues related to the topics under investigation had been reported by the interviewees during the last three interviews [29]. The remaining 6 OSWs and 5 patients who had already volunteered were interviewed. No new themes or issues were reported in these interviews confirming saturation of data. The OSW and patient interviews were analysed separately, using a thematic approach [30,31]. This was ongoing and iterative, such that analysis of early interviews informed the content of future interviews to ensure sufficient depth was reached. Analysis was carried out by AT. Three of the early interview transcripts from each interview set were coded independently by a second researcher (JM and FD) to ensure validity of coding categories and analytic rigour. The codes were compared by AT and found to have very close agreement and were used in the coding of subsequent interviews. Atlas.ti software [32] was used to facilitate coding, link codes and themes across interviews, and consider each theme in the context of the interview set. Since common themes arose in the OSW and patient interviews the results are presented together. Where a theme only arose or was more strongly emphasised in one or other set of interviews this is clearly stated. Illustrative quotes are included in figures to supplement narrative descriptions.

## Results

**Figure 1:**
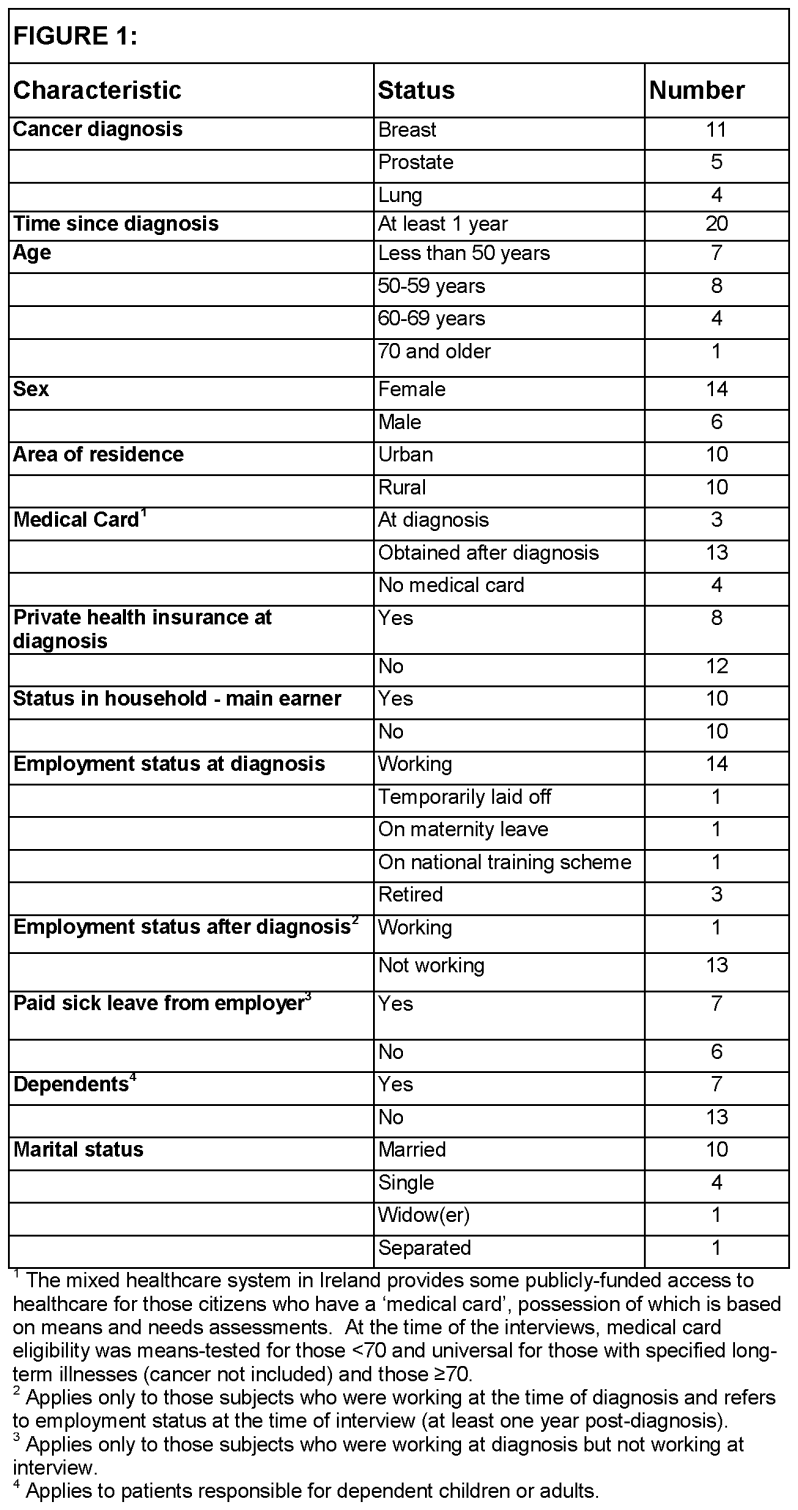
Characteristics of patients interviewed.

Twenty patients treated in 8 hospitals were interviewed (Figure 1). Twenty-one OSWs, from

**Figure 2:**
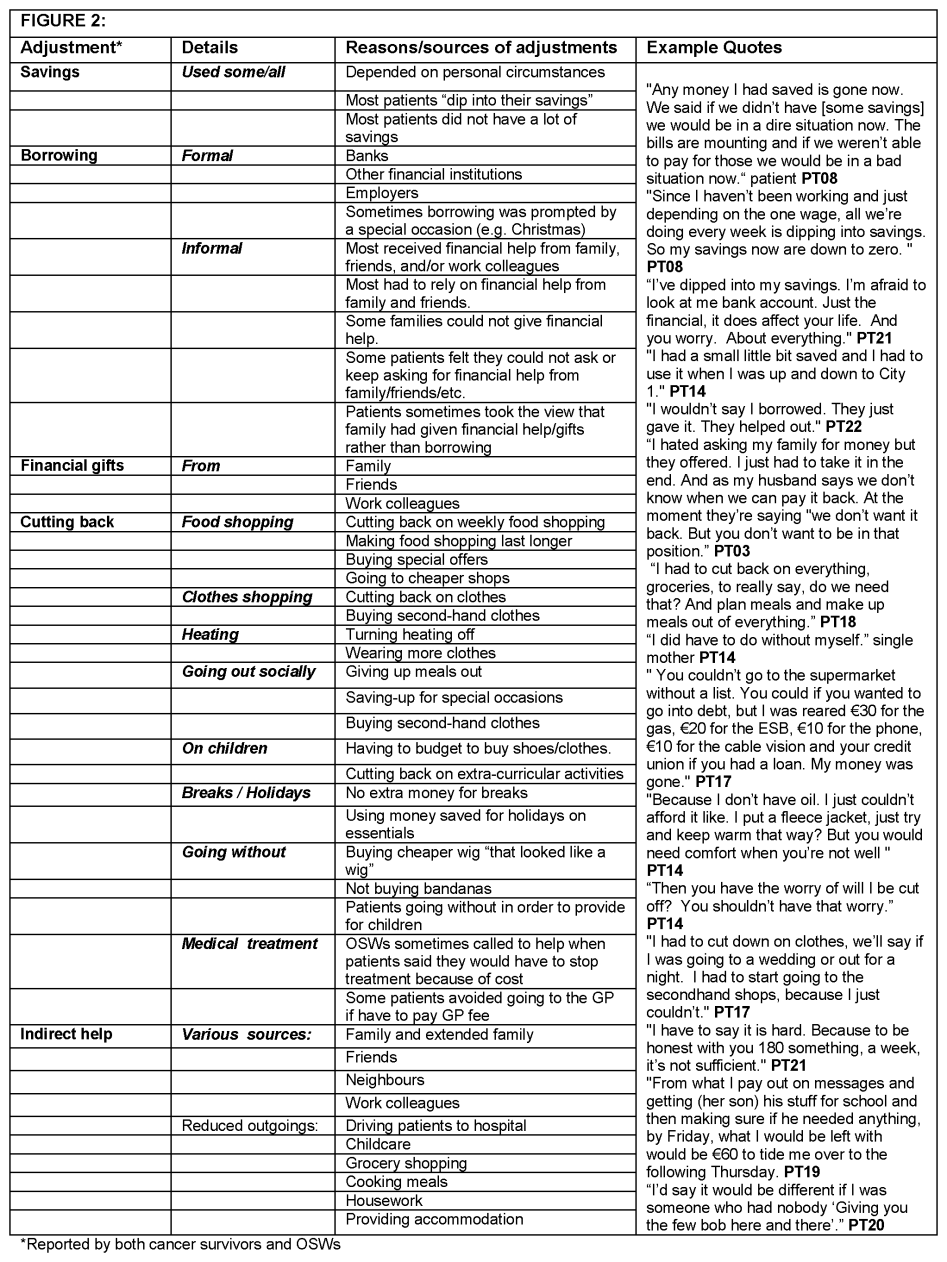
Financial adjustments.

**Figure 3:**
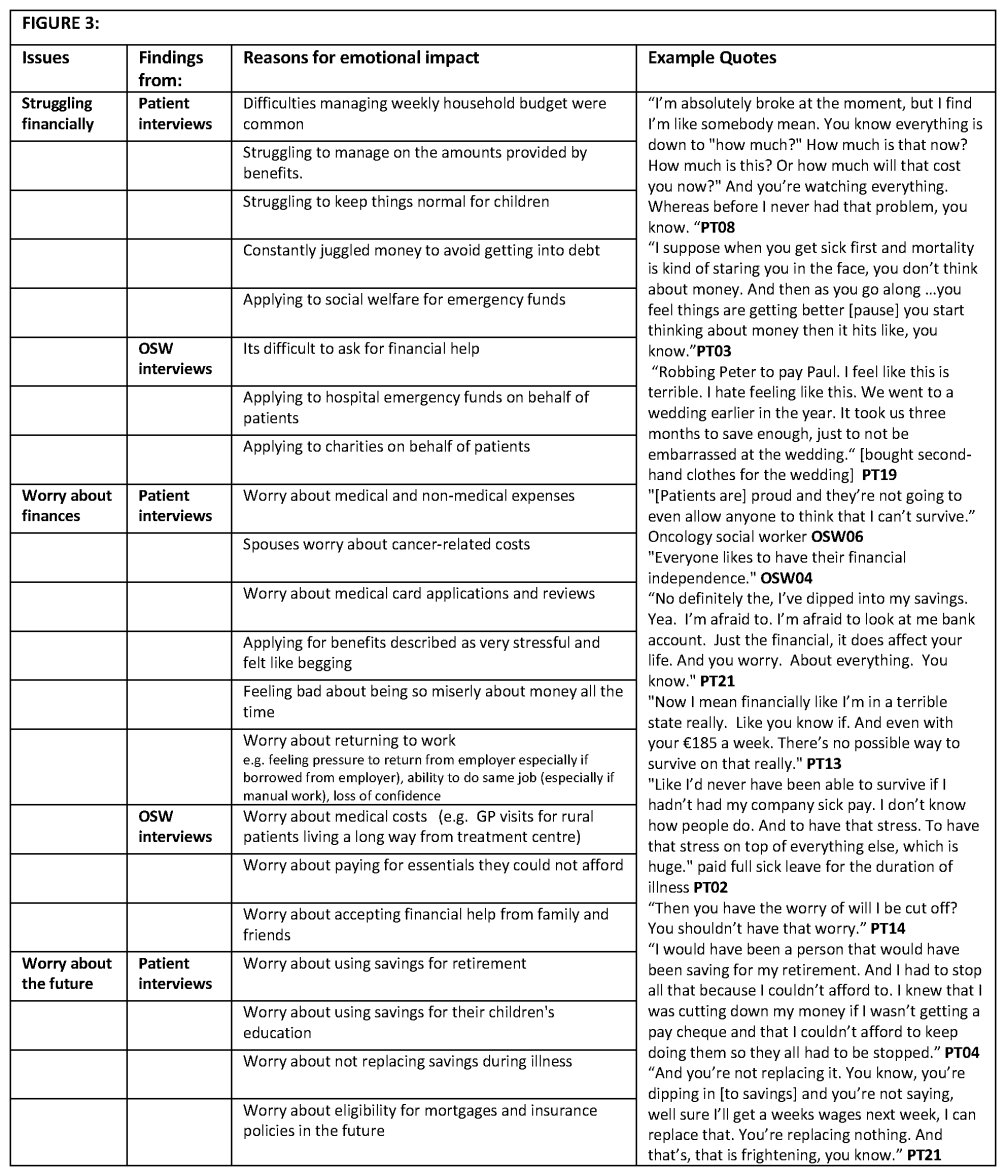
Social and emotional impact of financial burden of cancer.

**Figure 4:**
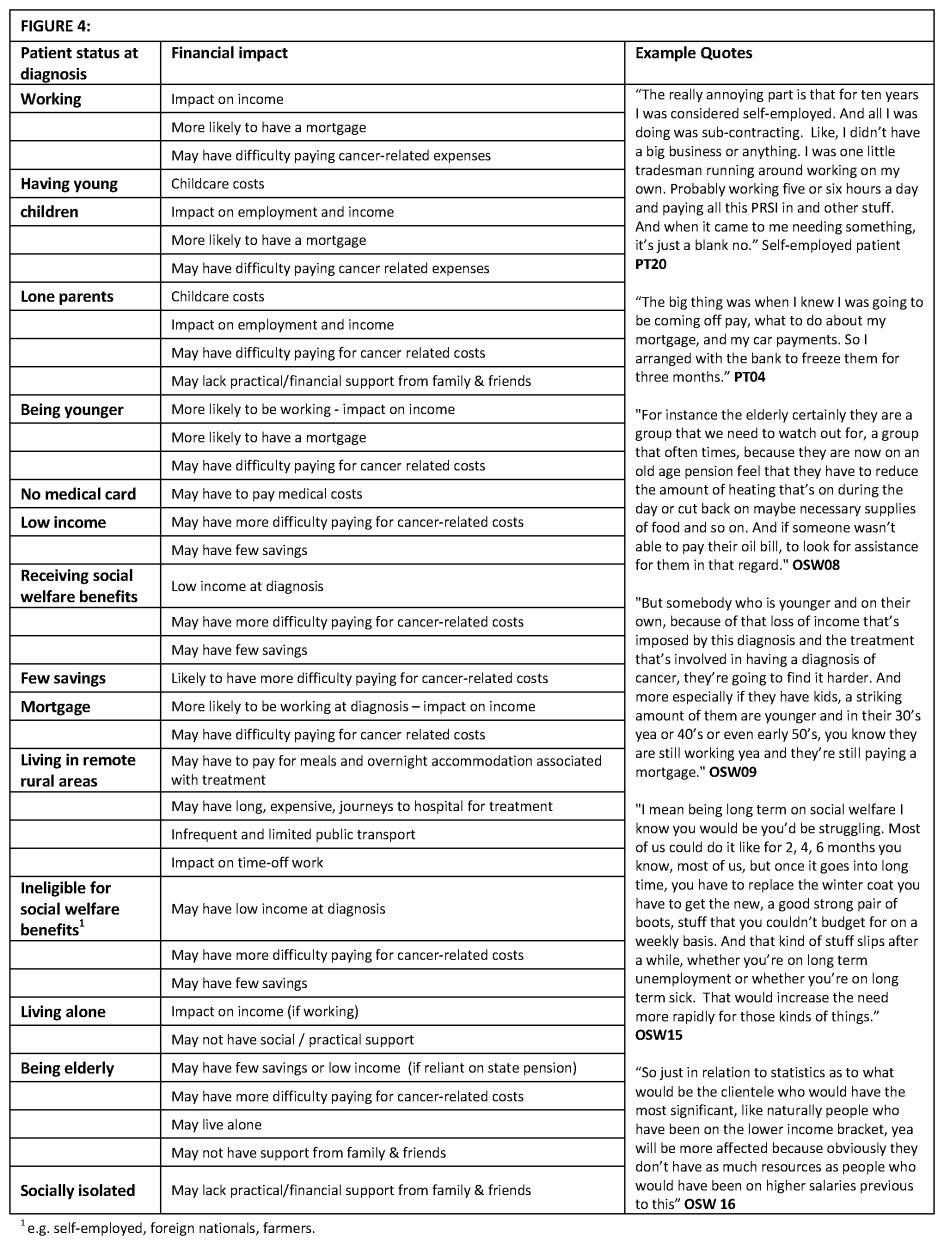
Factors increasing financial vulnerability.

11 hospitals, and with varying levels of experience, participated. Descriptions of OSWs’ characteristics have been limited to protect their anonymity. Three major themes relating to the changes to the financial situation experienced by patients after a cancer diagnosis were identified: financial adjustments needed (Figure 2); emotional consequences of these (financial distress) (Figure 3); and possible risk factors for financial difficulties and financial distress (Figure 4).

### Financial adjustments

All of the patients who stopped working after diagnosis or during treatment (Figure 1) experienced a drop in income even after compensatory payments (i.e. sick pay or health/social welfare benefits). All patients on a reduced income due to cancer, and patients with a low income pre-diagnosis (e.g. either earned or through pensions/benefits) found extra cancer-related costs difficult to manage. Patients and OSW reported that patients used a range of financial adjustments to try to cope financially (Figure 2).

#### Using savings, borrowing, and financial support from family and friends

Patients spoke about having to use some or all of any savings to help cope financially although some, particularly those with a low pre-diagnosis income, had few savings. Patients described borrowing money from various sources including banks/credit unions, employers, family and friends. Borrowing was often needed for special occasions (e.g. Christmas). Most patients reported having received some financial help from family, friends and/or work colleagues; this was not always perceived as “borrowing”, but rather as “help that was offered”.

#### Indirect financial support from family and friends

Patients often received other types of indirect financial support from family, friends, neighbours, and colleagues (e.g. driving to hospital appointments, childcare, grocery shopping, cooking meals, housework); these reduced the monetary costs associated with cancer. The security of having supportive family and friends was commonly spoken about; those who had support felt fortunate, but questioned whether it was “right” that they had to rely on this.

#### Cutting back on household spending

Patients reported struggling financially. Difficulties managing the weekly household budget were common. All patients except two (PT02 and PT10 discussed below) had to budget and spend more carefully following diagnosis. Strategies used included cutting back on food shopping; making food last longer; buying second-hand clothes; turning off heating more; socialising less often; not going to the hairdresser; reducing spending on children or “going without” themselves to provide for children; and not taking short breaks or holidays. Patients reliant solely on social welfare benefits experienced difficulties managing on the amount provided (€186/week). Large ongoing expenses (e.g. treatment-related travel costs, household bills) or one-off expenses (e.g. a new bed) were especially difficult for these patients. Some patients (particularly those working at diagnosis whose income fell) found the financial burden most difficult soon after diagnosis, whereas others (particularly those on a low pre-diagnosis income) found it more difficult as time went on.

#### Other sources of financial help

Some patients applied for means-tested financial assistance. For individual patients who were struggling financially, OSWs routinely applied for one-off welfare grants or financial assistance from charities.

#### Impact on patient care

OSWs reported sometimes being asked to help patients who were struggling financially to access financial assistance to help them comply with treatment. Examples included patients who were worried about being able to afford to buy medications or considering stopping treatment because they could not afford it. The OSWs reported that some patients (particularly rural patients living a long way from the treating centre) sometimes had to decide whether they would sit at home worrying rather than going to their GP about side-effects of treatment because of cost, or worry about spending money they couldn’t afford. One OSW described a patient who had postponed treatment until she received a medical card. A number of patients described how stressful hospital bills were while they were waiting for a medical card. None of the patients interviewed reported making treatment decisions based on cost/affordability.

### Financial distress

OSWs and patients described the emotional impact of the cancer-related financial impact (i.e. financial distress; Figure 3).

#### Financial worries

Medical bills, frequent reviews of medical card eligibility, and non-medical costs, in particular travel costs and increased household bills, caused considerable concern and worry for some patients. Patients described “scrimping and saving”, “robbing Peter to pay Paul” and feeling: “terrible”, “miserable”, or “horrible” about finances as well as feeling “like somebody mean” or “a real miser”. They described constantly juggling money to avoid getting into debt and emphasized how worried they were about their financial situation. Some patients who had stopped working during treatment were concerned about managing financially on sick pay and/or benefits. Patients felt that during treatment “their minds should be focussed on making themselves better and not worrying about finances”. OSWs reported that patients were generally uncomfortable with asking for, or accepting help, particularly from charities, and this caused stress and worry. They emphasised patient “vulnerability and how difficult it was for patients “to fight this [obtaining a medical card or benefits] themselves” after a cancer diagnosis. OSWs reported trying to help patients reduce financial stress but noted that because there were so few OSWs they could not help every patient.

#### Worries about the future

As well as being worried about their current situation, patients described worries about the future. These included worries about: not being able to replenish savings used during treatment/illness; implications of using money saved for retirement or children’s education; and paying back financial help received from family, friends, or employers. Younger patients described worrying about making mortgage payments and their future eligibility for mortgages and insurance policies. All patients who had been working at diagnosis expressed work-related concerns including: not feeling well enough to resume working; worry about returning to work or looking for a new job after a long absence; and loss of confidence. Impact of treatment side effects on work ability was a particular worry and cause of stress for patients who had developed lymphodema or nerve damage after surgery and had manual jobs.

### Possible risk factors for financial difficulties

OSWs reported that patients in almost any socio-demographic group could experience financial difficulties, depending on their particular circumstances and the support available to them. OSWs identified various possible risk factors for financial difficulties: working at diagnosis; having young children; being a lone parent; not having a medical card; low income pre-diagnosis (earned or through benefits); having no/few savings; having a mortgage; living in remote/rural areas without transport; being ineligible for means-tested benefits; living alone; and lack of family/social support (Figure 4). OSWs noted that not all patients in these groups experienced financial difficulties and risk factors often combined to influence patient vulnerability to cancer-related financial difficulties (e.g. low-income and few savings; working pre-diagnosis, with children and a mortgage; elderly with little social support). The patient experiences mirrored the OSW findings.

In contrast, OSWs reported that various factors could protect patients from financial difficulties post-cancer: receiving inpatient treatment; access to hospital accommodation (e.g. during radiotherapy); supportive family and friends able to provide financial or practical help; receiving full sick pay; and having other financial resources (e.g. savings, private health insurance, illness insurance). Again, combinations of factors meant that some patients did not have to make any financial adjustments. For example, a single breast cancer patient (PT02) received full sick pay, had private health insurance, and obtained a medical card post-diagnosis. A retired married prostate cancer patient (PT10) had surgery, few side-effects, private health insurance, and was eligible for a medical card. Neither of these patients reported any financial distress.

## Discussion

In the Irish mixed public-private healthcare system, we found that patients make a wide range of financial adjustments to cope with out-of-pocket costs and changes in income (financial burden) they experience post-cancer. Using savings, borrowing money (both formal and informal), and adjusting the weekly budget have been reported in a few previous studies in mainly private or mainly public healthcare settings [4,6,22,33–36]. The extent of financial adjustments needed varies in all settings but more extreme measures such a refinancing homes [37] or house repossessions [23] have been reported in both the US and the UK. Forgoing treatment or rationing medications because of cost has been described in other settings [6,38–42] although Housser et al. [43] recently reported that care-related cost savings were generally rare among breast and prostate patients in Canada. In our setting, patients were sometimes referred to OSWs to avoid this but none of the patients interviewed reported making treatment decisions based on cost/affordability. This may reflect the fact that this was uncommon or the fact that our study was conducted during an economic boom. As regards more novel findings, borrowing from employers does not appear to have been reported elsewhere and may be related to the limited provision of sick pay in Ireland (and may also apply in other settings where sick pay provision is scanty). Informal financial support from family or friends also emerged in a study of colorectal cancer in Ireland [44]. In our study patients did not always consider this “borrowing”, which suggests that quantitative studies may underestimate the prevalence of borrowing money to cope financially post-cancer. 

It is clear from our study, and qualitative research in the UK and North America [6,22,23,45] that the cancer-related financial burden and financial adjustments can affect patients (and their families) emotionally and psychologically. This is important because cancer-related financial stress (financial stress experienced post-cancer due to events that are financial stressors for the household) and financial strain (an individual’s subjective reactionary perception of financial stress experienced) have been found to be independent predictors of psychological well-being post-cancer [46]. Similarly, financial distress has been reported in cancer patients with health insurance in the US [37]. Financial distress is itself associated with negative health effects, including anxiety, insomnia, headaches and depression [17]. It is possible that financial distress may extend the duration of cancer’s financial impact by delaying the time to recovery. Bradley [47] recently highlighted the need for a better understanding of how financial hardship varies across patient groups suggesting this might provide insight into sources of disparity and how they might be mitigated. Some of the patient characteristics identified by OSWs as possible risk factors for inadequate financial protection also emerged from a few studies of other cancers in other settings. These include: low income [1,5,37,42,48]; being employed or self-employed [4,49]; being younger [1,7,37,48]; having children [50]; and living in rural areas or further from treating centre [33,48]. Where our findings extend those from other studies is that they suggest that it is likely that a combination of risk factors operate together to influence financial protection - either negatively or positively. The latter is important in better understanding what may protect patients from financial difficulties post-cancer.

### Implications for financial protection in health

Clearly in addition to patients’ individual circumstances (e.g. availability of savings/assets, financial support from family/friends) and the financial compensation and protection provided by the public or private components of the healthcare system, the legal and social welfare system where patients live is also important in influencing the financial impact of having cancer. However, despite international variations in healthcare, legal and social welfare systems, our findings add to those from elsewhere [5,23,39,40] to suggest that – in all (or, at least, almost all) settings - some cancer patients need to make financial adjustments and have inadequate financial protection post-cancer. Furthermore, findings from the current study point to the fact that the financial impact of cancer, and inadequate financial protection, affects the whole family unit and not just the patient themselves. Several authors have called for more sensitive and standardised ways of capturing cancer or illness-related costs to patients and families [15,47,51]. Financial adjustments and financial distress are rarely considered in formal assessments of the financial/economic impact of cancer either in research or the practice/clinical setting. Nor are they included in conventional frameworks for analysing financial protection in health. Both Moreno-Serra et al. [15] and Ruger [20] have recently suggested that current measures of the consequences of inadequate financial protection are too narrow and probably underestimate the adverse consequences of inadequate financial protection in health. Ruger [20] has proposed an alternative multidimensional approach using a “financial protection profile” to better reflect how individuals and households cope financially after confronting a health need. Our findings reported here and elsewhere [9] also highlight the need for more comprehensive metrics to assess financial protection in health, which we suggest should include: out-of-pocket direct medical and non-medical costs; changes in income (which are not included in Ruger’s financial protection profile); financial adjustments (including coping strategies and household consumption patterns as per Ruger); and financial distress (not included by Ruger).

### Implications for financial information and advice services

The need for financial assessment, information, and advice intermittently throughout the cancer journey has been highlighted both in Ireland and the UK [9,52,53]. In the UK, Moffatt et al. [54], recently demonstrated the benefits of a welfare rights advice intervention designed to address the financial consequences of cancer. Importantly, in that study, and in a US study on the effects of participation in a debt management programme [17], as well as financial benefits, these initiatives resulted in positive social and psychological consequences; this suggests that interventions aimed at alleviating financial burden and adjustments post-cancer may also alleviate financial distress.

### Strengths and limitations

The use of explorative qualitative methods has improved understanding of the complex financial adjustments cancer patients use to cope with their financial situation and, importantly, the consequences of these (i.e. financial distress). Data of such richness and depth would not have been available using a quantitative approach. While it is not possible to estimate the proportion of cancer patients with financial difficulties that discussed their financial situation with an OSW, the inclusion of 87% of the OSWs in the country who collectively have assisted large numbers of cancer patients over many years, corroborated the information from the patient interviews, and provided valuable additional insight into the predictors of financial adjustments and financial distress. This important “bigger picture” would not have been evident from patient interviews alone. Moreover, our results extend the existing evidence-base on the financial effects of cancer by adding data from a mixed public-private healthcare system. The fact that the healthcare systems in most countries are based on a combination of libertarian (private) and egalitarian (public) ideologies [21] suggests that the results of this study may have broader implications for other jurisdictions. We explicitly included patients who had reported experiencing “extra costs” or “financial difficulties” due to cancer and this enabled us to capture the multidimensional aspects of the financial impact. To achieve a maximum variation patient sample we relied on OSWs and oncology nurses to identify potential participants, using stratification based on gender, age, employment status, cancer type, and residence. While professionals’ role in identification of participants was critical to ensure that sharing of patient information with the researcher team took place on an ‘opt-in’ basis (in line with good practice), this did mean that there was a possibility that professionals selected those patients who they thought would represent particular perspectives. While it is certainly possible that professionals were thoughtful about their selection of patients, the researcher discussed with them the need for a diverse sample prior to and during the recruitment process. In addition, the credibility of the research processes, including the sampling, is evidenced by the varied experiences and opinions contained in the dataset as a whole.

## Conclusions

 Both patients and OSWs reported that some patients need to make financial adjustments in order to cope financially after a cancer diagnosis. A broad range of financial adjustments were reported and included: using savings (if any); formal and informal (from family and friends) borrowing (if available); budgeting very carefully; and cutting back on weekly household spending. Moreover, financial distress is a common outcome of cancer-related out-of-pocket costs, lost income and these financial adjustments. Our findings add to an accumulating evidence-base which implies that the different types of healthcare system (mixed public/private, mainly public, and mainly private) may not provide financial protection for patients and families post-cancer. To advance understanding, and inform development of effective strategies, interventions or policies, there is a need for a broader and more comprehensive set of metrics to measure financial protection in health; these should include consideration of financial adjustments and financial distress. In the meantime, patients and their families should be offered financial information and advice intermittently throughout the cancer journey.
